# Independent Aftereffects of Fat and Muscle: Implications for neural encoding, body space representation, and body image disturbance

**DOI:** 10.1038/srep40392

**Published:** 2017-01-10

**Authors:** Daniel Sturman, Ian D. Stephen, Jonathan Mond, Richard J Stevenson, Kevin R. Brooks

**Affiliations:** 1 Department of Psychology, Macquarie University, Sydney, Australia; 2 ARC Centre of Excellence in Cognition and its Disorders, Macquarie University, Sydney, Australia; 3 Perception in Action Research Centre (PARC), Faculty of Human Sciences, Macquarie University, Sydney, Australia; 4 Centre for Health Research, School of Medicine, Western Sydney University, Sydney Australia; 5 Centre for Rural Health, University of Tasmania, Launceston Tasmania

## Abstract

Although research addressing body size misperception has focused on socio-cognitive processes, such as internalization of the “ideal” images of bodies in the media, the perceptual basis of this phenomenon remains largely unknown. Further, most studies focus on body size *per se* even though this depends on both fat and muscle mass – variables that have very different relationships with health. We tested visual adaptation as a mechanism for inducing body fat and muscle mass misperception, and assessed whether these two dimensions of body space are processed independently. Observers manipulated the apparent fat and muscle mass of bodies to make them appear “normal” before and after inspecting images from one of four adaptation conditions (increased fat/decreased fat/increased muscle/decreased muscle). Exposure resulted in a shift in the point of subjective normality in the direction of the adapting images along the relevant (fat or muscle) axis, suggesting that the neural mechanisms involved in body fat and muscle perception are independent. This supports the viability of adaptation as a model of real-world body size misperception, and extends its applicability to clinical manifestations of body image disturbance that entail not only preoccupation with thinness (e.g., anorexia nervosa) but also with muscularity (e.g., muscle dysmorphia).

Body size misperception is a phenomenon wherein people believe themselves or others to be larger or smaller than they actually are^1^,^2^,^3^. In research conducted in a broad range of populations, and in both adolescents and adults, as many as half of participants have been found to misperceive their body weight^4^,^5^,^6^,^7^,^8^.

This is concerning in two respects^3^. First, individuals who are underweight or normal-weight according to accepted classifications but who believe themselves to be overweight are likely to have high levels of body dissatisfaction and, in turn, increased risk of mental health problems such as eating disorders, anxiety and depression^9^,^10^,^11^. Further, a recent study involving a multisensory illusion induced using a virtual reality set-up has shown a causal link between changes in perceived body size and changes in body dissatisfaction^12^. Conversely, individuals who are overweight or obese but who believe themselves to be of normal or otherwise acceptable weight may be less motivated to make efforts to reduce body weight, and less likely to seek help for obesity-related medical problems^6^,^8^,^13^.

To date, research addressing body size misperception has tended to focus on socio-cognitive processes, such as internalization of the “ideal” images of male and female bodies portrayed in the popular media^7^,^14^,^15^ or, in the case of underestimation of body size among overweight individuals, increased exposure to obesity in everyday life and a consequent change in what is considered a “normal and healthy” body weight^3^,^8^,^16^. As a consequence, little is known about the perceptual mechanisms underpinning body size misperception. However, there is growing evidence that the relationship between exposure to different bodies and body size misperception may be mediated by a perceptual adaptation effect. While the early literature concentrated on linking examples of media exposure and body size misperception outside the laboratory, more recent experimental studies have established a causal link under controlled conditions^17^,^18^,^19^,^20^,^21^.

Adaptation has been extensively examined in visual perception, wherein extended exposure to a particular image (the “adaptation” stimulus) causes a visual aftereffect: a bias in the observer’s perception of similar “test” stimuli^22^. For example, viewing a pattern of thin vertical stripes for 30 seconds causes a set of broader stripes to appear even wider than they really are, and vice versa – the well-known spatial frequency aftereffect^23^,^24^. By examining the magnitude of aftereffects following exposure to various adaptation and test stimuli, this technique provides a non-invasive means of studying the neural mechanisms responsible for their processing^25^.

Early work on visual adaptation included simple stimuli differing in terms of their basic perceptual properties, such as direction of motion, colour, orientation and spatial scale^26^. More recently, researchers have begun to examine the aftereffects that occur in more complex stimuli such as faces, which are believed to result from adaptation to higher-level neural structures. In face perception, aftereffects have been found to occur with facial expressions^27^, age^28^, sex and race^29^, and even for geometrical deformations such as expansion or contraction^30^,^31^,^32^. The psychological explanation for these higher level effects is usually couched in terms of changes to the cognitive representation^33^, or to the updating of perceptual “norms”^34^, formed from the average of recently encountered faces. In general, the stimuli to which the participant is exposed are incorporated into the pool of faces that are averaged, making the average more similar to those faces. For example, when an expanded face is viewed for an extended period, the observer’s norm is temporarily shifted such that these stimuli are perceived as less unusual or extreme. Consequently, a face that previously appeared normal now appears contracted. This norm shift is thought to be the result of the reduction of sensitivity that is known to result from prolonged stimulation of cells in the visual system^35^,^36^.

As with faces, body aftereffects have been demonstrated along several dimensions including size^21^,^37^, gender^38^,^39^, viewpoint and pose^40^. As with face distortion aftereffects, adaptation to images of bodies that have been compressed or stretched horizontally to simulate smaller or larger body sizes results in perceptions of what is average shifting to appear more similar to the adaptation images^21^, along with effects on judgements of attractiveness^41^,^42^,^43^,^44^. This effect has been replicated using more realistic computer generated bodies^18^ and real photographs^36^. Importantly, researchers have demonstrated that body size aftereffects can transfer between adaptation images of other individuals and test images depicting one’s self^17^,^19^,^37^. This kind of cross-adaptation is necessary if the mechanism of adaptation is to provide an explanation for real-world examples of body size misperception and the link with exposure to images of thin models in the media.

The majority of body adaptation experiments to date have focused on a simple concept of body size. However, bodies typically vary in terms of their levels of fat and muscle, which together form the two-dimensional concept of body composition. While both of these dimensions have a positive relationship with body size, muscle and fat have opposite relationships with health. Generally, increased body fat has a negative impact on health^45^,^46^, while the effect of increased muscle mass is positive^47^. As the health risks associated with being overweight are associated with high fat, not muscle, body composition is arguably a more important measure than simple body size^48^,^49^.

Body composition is a crucial concept in understanding body size misperception, its associations with body dissatisfaction and the sex differences in these associations. In females, body dissatisfaction tends to relate to the amount and distribution of body fat^50^, whereas in males it typically relates to a desire for both less fat and greater muscle mass^51^. Further, body fat dissatisfaction and muscle dissatisfaction, in their extreme forms, appear to be associated with distinct, though related, forms of psychopathology^51^,^52^. Thus, individuals (typically males) with the body image disorder muscle dysmorphia tend to misperceive their muscularity but not their level of body fat^53^,^54^, while individuals (typically females) with *anorexia nervosa* have been found to misperceive their levels of body fat, not muscle mass^55^,^56^.

If the examples of body size misperception can be considered to be real-world examples of visual adaptation, as suggested by previous experimental studies, then this technique should be capable of producing independent effects of exposure to fat and muscle. Such a result would reflect independent neural mechanisms for these two stimulus attributes, and the shifting of norms specifically along separate dimensions of body space. To establish whether or not this is the case, this study will adapt observers to photorealistic images of bodies that have a high or a low level of body fat or muscle mass and assess the resulting perceptual aftereffect specifically in terms of these two independent dimensions. If the perception of body fat and muscle mass is confounded by a single neural mechanism encoding body size, exposure to bodies with high or low levels of body fat should alter perceptions of both body fat and muscle mass, as should exposure to muscular bodies. However, if body size is encoded independently along the dimensions of muscle mass and body fat, then it is predicted that prolonged exposure to bodies with high or low body fat should alter perceptions of body fat, but not muscle mass, and *vice versa*. This question has not previously been examined.

## Methods

All methods were performed in accordance with the relevant guidelines and regulations, and were approved by the Macquarie University Human Research Ethics Committee.

### Participants

Stimulus collection involved 192 Caucasians (64 males) between the ages of 18 and 30 years (*M* = 20.76 years, *SD* = 5.35). These photographic subjects did not participate further. Following this, perceptual judgements were made by 64 observers (25 males). All had normal or corrected to normal vision, were over 18 years of age (*M* = 22.62 years, *SD* = 4.81) and were naïve to the experimental hypotheses. All participants were recruited from a UG Sample at Macquarie University, gave prior informed consent in writing, and participated for course credit or compensation of $20.

### Stimuli

Photographs were taken of subjects wearing standardised tight fitting grey singlets and shorts, posed in a standardised anatomical position from a frontal view^41^,^57^,^58^. Photographs were taken inside a booth that was painted in Munsell N5 neutral grey, and illuminated with 15 high accuracy d65 fluorescent Philips tubes in high frequency fixtures to reduce the effects of flicker. A Canon 50D digital camera was used, with exposure, ISO and custom white balance held constant. A Tanita SC 330 body composition analyser was used to measure weight, body fat and muscle mass, and a fixed measuring tape was used to measure height. This device measures body fat and muscle mass using the Bioelectrical Impedance method, *which has an accuracy of* ±*2%, and gives readings up to a precision of 0.1 kg*^59^.

Images of male and female subjects were treated separately. The background of all images was rendered a uniform grey, and Psychomorph was used to align stimuli to remove translational and rotational variation between images. Stimuli were then manipulated independently in terms of their apparent body fat and muscle mass using Psychomorph version 6^60^. For each image, 130 landmark points were marked to delineate the features of the body. Regression analyses were used to calculate a standardised body fat score for each body that controlled for muscle mass and height, and a standardised muscle mass score that controlled for body fat and height. Images with muscle mass more than 1 standard deviation from the mean were temporarily removed, after which the images were ranked by standardised body fat scores. The 10 highest ranked images for each sex were grouped, and the location of each landmark point was averaged to create a ‘high fat’ prototype for each sex, while the lowest 10 were averaged to create a ‘low fat’ prototype. The same process was repeated using muscle mass scores to create the ‘high muscle’ and ‘low muscle’ prototypes for each sex. However, due to the smaller sample of males with extreme muscle and average fat, only 5 images were used for the male ‘muscle’ prototypes. The difference in mean body fat and muscle mass for each prototype can be seen in Table 1. Independent samples t-tests showed significant differences in fat levels, but not muscle levels, for the ‘fat’ prototypes, and significant differences in muscle levels, but not fat levels, for the ‘muscle’ prototypes.

These prototype images allowed us to define the image transformations that accompany increases or decreases in body fat or muscle independently of any other aspects of the image, using Psychomorph. Twenty-five male and 25 female images with both body fat and muscle mass scores less than 1 standard deviation from the mean that were not used in the creation of the prototypes were selected as test identities for fat and muscle transformations. Each image was manipulated in 13 equidistant steps along the body fat dimension from a manipulation of 100% of the difference between the prototypes towards the low fat prototype and ending with a 100% manipulation towards the high fat prototype.

**Figure 1:**
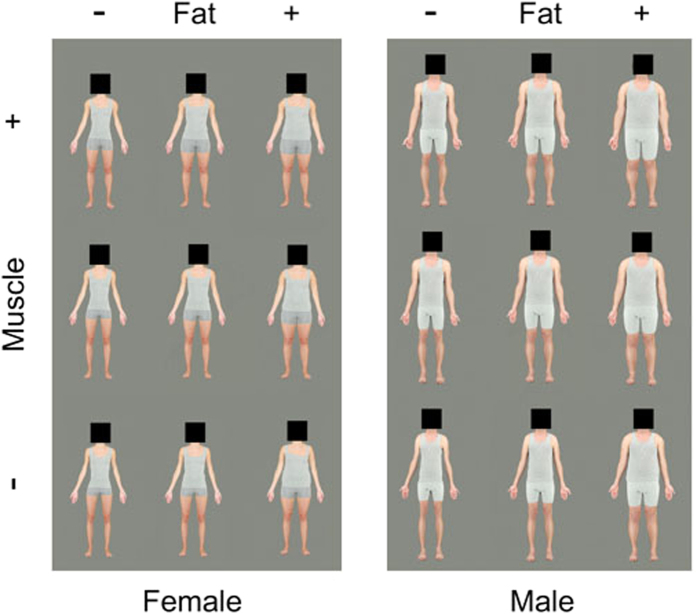
Examples of a female and a male body manipulated along the body fat and muscle mass dimensions, with the original image (centre) and 100% manipulations towards the low fat, high fat, low muscle and high muscle prototypes.

Each of the 13 images were then individually manipulated in 13 equidistant steps along the muscle mass dimension from 100% of the difference between the prototypes towards the low muscle prototype and ending with a 100% manipulation towards the high muscle prototype. This resulted in a two-dimensional grid of images that increased incrementally by 13 steps along the body fat dimension and 13 steps along the muscle mass dimension, resulting in a set of 169 images for each test identity (see end-points in Fig. 1).

Images were formatted to 600 × 900 pixels for use as test stimuli and 450 × 675 pixels for use as adaptation stimuli to avoid low-level retinotopic adaptation aftereffects. The face of each image was obscured with a black square. For each observer, five identities were randomly selected as practice identities, 10 identities were randomly selected as test stimuli and the remaining 10 identities were used as the adaptation stimuli.

A body manipulation tool, created using Matlab, divided the monitor into an invisible grid of 169 cells (13 cells across and 13 cells high), which correspond to the 169 images for each identity. When the mouse cursor, which was not visible to the observers, was placed over a cell the image with the corresponding fat and muscle score was displayed on the monitor. This allowed for independent manipulation of the dimensions of body fat and muscle mass using horizontal and vertical mouse movements.

### Procedure

Observers were randomly assigned to one of four adaptation conditions; low fat, high fat, low muscle or high muscle. The testing procedure consisted of 3 blocks; practice, baseline testing and adaptation testing. Images were presented in Matlab version 8.3 (R2014a) and viewed on a 19” Samsung 943BW colour monitor (resolution 1600 × 900 pixels) at a distance of 57 cm.

Observers were then presented with a practice identity and, using the body manipulation tool, were instructed to manipulate the body image until it represented an ‘average sized body’, and to select this image with a mouse click. This process continued until all 5 practice identities had been presented.

Baseline testing was then carried out using the same instructions and procedures as those used in the practice phase. Observers were required to manipulate each of the 10 test identities, which were presented in a pseudo-random order, twice. For each observer, a baseline point of subjective normality (PSN) was calculated for body fat (PSN_f_) and for muscle mass (PSN_m_) by averaging their fat scores and muscle scores respectively.

The adaptation test block was identical to the baseline test block, except for the exposure to adaptation stimuli. “Initial” adaptation preceded the manipulation of any test stimuli: the 10 adaptation stimuli were presented 6 times each for 2 seconds in a random order (total 120 s adaptation time). Top-up adaptation occurred in between each test stimulus presentation, and consisted of 6 different identities randomly selected after each trial from those used in the adaptation phase, each presented for 1 second. The level of fat and muscle displayed in the adaptation stimuli were based on the observer’s baseline PSN_f_ and PSN_m_ scores. Those in the high and low fat conditions were presented with adaptation images that were 6 image steps higher (to a maximum of 100%) or lower (to a minimum of −100%) than their baseline PSN_f_, and those in the high and low muscle conditions were given adaptation images 6 steps higher or lower than their baseline PSN_m_.

The entire procedure was completed twice, once using male and once using female stimuli, in a counterbalanced order.

### Statistical analysis

Adaptation-induced changes in PSN_f_ (henceforth referred to as ∆PSN_f_) and change in PSN_m_ (∆PSN_m_) were calculated by subtracting the baseline PSN scores from adaption PSN scores, expressing the change as a percentage of the extremity of the adaptation image. Positive ∆PSN_f_ or ∆PSN_m_ scores corresponded with aftereffects in which perceptions of average body fat or muscle mass increased following adaptation, while negative scores corresponded with aftereffects in which perceptions of average decreased following adaptation. Data were analysed using a 2 × 2 between subjects ANOVA for each of the dependent variables. The between subjects variables were adaptation direction (increase/decrease) and adaptation dimension (body fat/muscle mass).

## Results

**Figure 2:**
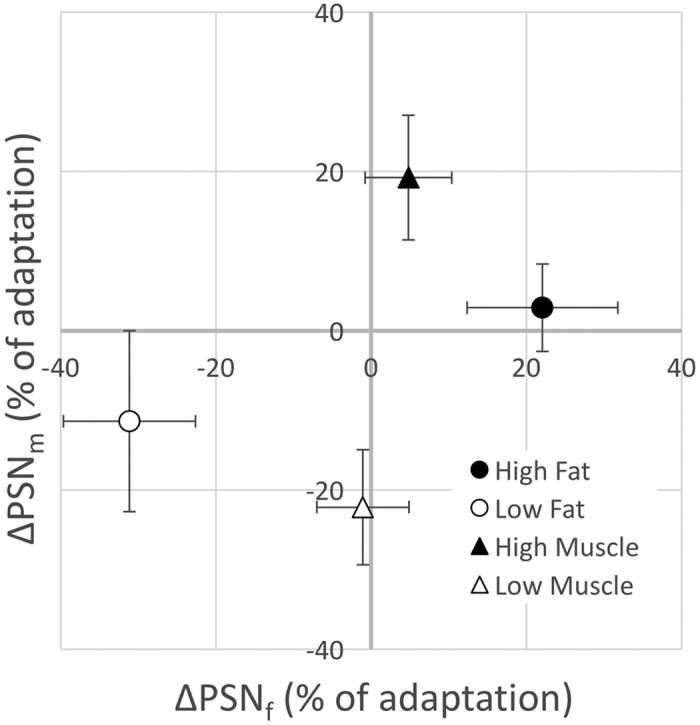
Mean ∆PSN_f_ (left) and ∆PSN_m_ (right) following adaptation. Error bars represent 95% confidence intervals.

As expected, the main effect of adaptation direction was significant when considering both perceived fat and perceived muscle (see Fig. 2). The fat ANOVA revealed a significantly more positive ∆PSN_f_ for observers who adapted to images with increased body size (*M* = 13.5, *SE* = 2.5), compared to those who adapted to images with decreased body size (*M* = −16.2, *SE* = 2.5), *F*(1,60) = 67.91, *p* < 0.0005, η_p_^2^ = 0.53. A similar effect was observed when analysing the effects on perceived muscle mass, where the muscle ANOVA demonstrated a significantly more positive ∆PSN_m_ for observers who adapted to images with increased body size (*M* = 11.0, *SE* = 2.6) compared to observers who adapted to images with decreased body size (*M* = −16.8, *SE* = 2.7), *F*(1,60) = 51.59, *p* < 0.0005 η_p_^2^ = 0.46. As expected, neither ANOVA revealed a statistically significant main effect of adaptation dimension (*p* > 0.05).

In addition, a significant interaction between adaptation dimension and adaptation direction was observed for the fat ANOVA (see Fig. 2). The difference between ∆PSN_f_ for the high and low fat adaptation conditions was significantly greater than the difference between the high and low muscle adaptation conditions, *F*(1,60) = 43.55, *p* < 0.0005 η_p_^2^ = 0.42. Similarly, for the muscle ANOVA, the difference between ∆PSN_m_ scores for the high and low muscle conditions was significantly greater than the difference between the high and low fat conditions, *F*(1,60) = 12.24, *p* = 0.001, η_p_^2^ = 0.17.

Four planned comparisons were performed using independent samples t-tests (2-tailed), with an adjusted individual comparison alpha level of 0.0125 (i.e. using Dunn’s test^61^ to maintain a family-wise error rate of α = 0.05). For body fat settings, mean ∆PSN_f_ was significantly more positive for the high fat adaptation condition (*M* = 22.1, *SE* = 4.6) than the low fat adaptation condition (*M* = −31.1, *SE* = 4.0), *t*(30) = 8.77, *p* < 0.0005, whereas there was no significant difference in mean ∆PSN_f_ between those in the high muscle adaptation condition (*M* = 4.8, *SE* = 2.6) and those in the low muscle adaptation condition (*M* = −1.1, *SE* = 2.8), *t*(30) = 1.54, *p* = 0.135. Considering muscle mass settings, mean ∆PSN_m_ was significantly more positive for those in the high muscle condition (*M* = 19.2, *SE* = 3.8) compared to those in the low muscle condition (*M* = −22.2, *SE* = 3.3), *t*(30) = 8.29, *p* < 0.0005. However, the difference in mean ∆PSN_m_ for those in the high fat condition (*M* = 2.8, *SE* = 2.5) compared to those in the low fat condition (*M* = −11.3, *SE* = 5.3), *t*(30) = 2.41, *p* = 0.022 was not significant.

A post-hoc test of the hypothesis that observers with extreme baseline PSNs on a particular dimension (fat or muscle) might show larger adaptation effects along that dimension (i.e. a positive correlation) was also conducted. Two-tailed Pearson product-moment correlation coefficients (r) were computed to assess the relationship between baseline PSN_f_ and ∆PSN_f_ for the high and low fat adaptation conditions, and between baseline PSN_m_ and ∆PSN_m_ for the high and low muscle adaptation conditions. While two conditions showed negative r values, there was no significant correlation between the two variables in any condition, even before considering any adjustment for multiple comparisons (high fat: r = 0.082, p = 0.763; low fat: r = −0.431, p = 0.096; high muscle: r = 0.186, p = 0.490; low muscle: r = −0.239, p = 0.373).

## Discussion

To investigate the mechanisms of perception for various aspects of human bodies, we used a visual adaptation paradigm, observing the aftereffects of exposure to specific body sizes and shapes. In line with our predictions, observers showed aftereffects of body size after prolonged viewing of large and small bodies (main effects of adaptation direction). These results are consistent with past research regarding body size adaptation, which found that size aftereffects occur such that the point of perceived normality shifts in the direction of the adapting bodies^17^,^18^,^20^,^21^,^36^,^37^.

To examine, for the first time, whether the dimensions of body fat and muscle mass are encoded by common or dissociable neural mechanisms, we observed the specific effects of each adaptation condition on perceived levels of fat and muscle. The hypothesis that dissociable neural mechanisms encode the separate body composition dimensions of body fat and muscle mass was supported, as the effect of adaptation on perceived body fat was significant when adaptation stimuli differed in their fat content but not when they differed in terms of muscle mass. Similarly, an aftereffect on perceived muscle mass was shown when adaptation stimuli involved unusual levels of muscle, but not when they involved unusual levels of fat. The adaptation effect caused by exposure to low fat images appeared to cause a different effect on perceived muscle mass than exposure to high fat images which would be significant in an uncorrected analysis. If this effect were considered significant, it would indicate a surprising asymmetry in the processing of muscle and fat. While bodies that are extreme in their body fat levels (particularly those on the low end of the scale) principally adapt neurons that are tuned to respond to fat, they also adapt muscle mechanisms to a small degree. Yet bodies that are low on muscle do not adapt fat mechanisms. However, after correction for multiple comparisons this effect was not found to be significant. In general, exposure to bodies with extreme fat levels causes fat aftereffects, and exposure to bodies with extreme muscle levels causes muscle aftereffects. This suggests that a multi-dimensional body space, similar to face space^62^ may be a useful framework for body perception and representation, and is consistent with the idea that adaptation causes norms to be updated, moving towards the adaptor along a given dimension.

The current study improves on the existing body adaptation literature by using stimuli that more accurately represent various body sizes and compositions. Previous studies manipulated images by stretching the entire image horizontally^21^, by expanding the image principally around the waist-hip region^17^, through the distortion of different body “zones”^36^, or by manipulating computer generated bodies^18^. Instead, we used advanced image morphing techniques to create more realistic manipulations. Furthermore, the photographic subjects came from a non-clinical sample and represented a normal range of body compositions. This improves the ecological validity of the current study, as stimuli are realistic and representative of the variation present in a normal population.

Importantly, these results may have implications for real world examples of body size misperception, including body image disturbance and associated pathologies. A visual adaptation model of body size misperception is consistent with the view that individuals who are of normal weight or underweight may misperceive themselves to be overweight if regularly exposed to low body fat models in the media^7^,^14^,^15^. For individuals who do not regularly encounter such images and who tend to interact with individuals who are overweight or obese, by contrast, an underestimation of body fat would be predicted^3^,^8^,^16^. Along similar lines, regular exposure – and hence adaptation – to highly muscular body shapes, in the context of body building training or competition for example, may cause individuals to believe that they are less muscular than they really are. In all of these cases, body size misperception may increase the risk of adverse physical and mental health outcomes^3^,^6^,^7^,^11^. While it may be premature to assert that visual adaptation underlies each of these phenomena, the current study, in concert with the previous literature, attests to the viability of explanations in terms of long-lasting visual aftereffects caused by the continual adaptation of the visual system to specific body types. In agreement with previous studies showing that the body size aftereffect transfers between images of self and other^17^,^37^, here, adaptation and test stimuli involve different bodies, showing that the independent fat and muscle aftereffects are not specific to particular images or identities. Further tests of the generality of this effect may involve images taken from different viewpoints, with different clothes, or under different lighting conditions. If the effects persist, the research may be advanced by considering how the adaptation paradigm might be used to reduce levels of body size misperception both in actual real-world settings.

Although it was not possible in this initial study, for which participants were recruited from a convenience sample, with small and unequal numbers of males and females in each group, it would be of interest to examine sex differences in fat- vs muscle-related adaptation effects in future research. Ideally, subgroup sample sizes would be sufficient to manipulate the sex of both photo participants and observers in order to determine the independent effects of both variables. Differences in the susceptibility of males and females to aftereffects from images that are extreme in terms of their fat or muscle content might be hypothesised, given the known sex differences in body image concerns relating to these different dimensions of body composition^43^,^44^. On the other hand, findings from recent studies suggest that both females and males are increasingly pursuing a body type characterised by both low levels of adiposity and high levels of muscularity^63^,^64^, such that differences in adaptation effects along these dimensions may be less apparent than expected.

In sum, this study is the first to demonstrate that adaptation can occur independently along the different dimensions of body composition. That aftereffects were restricted to the dimension in which the adaptation stimuli varied constitutes evidence for neural mechanisms that are independently sensitive to fat and muscle. The results confirm the viability of visual adaptation as a model of body size misperception and suggest that this model may be applicable to both thinness- and muscularity-oriented manifestations of body image disturbance.

## Additional Information

**How to cite this article:** Sturman, D. *et al*. Independent Aftereffects of Fat and Muscle: Implications for neural encoding, body space representation, and body image disturbance. *Sci. Rep.* **7**, 40392; doi: 10.1038/srep40392 (2017).

**Publisher's note:** Springer Nature remains neutral with regard to jurisdictional claims in published maps and institutional affiliations.

## Figures and Tables

**Table 1:** Comparisons of Body Composition between Prototype Images.

Prototypes	Fat difference (kg)	Muscle difference (kg)
High fat vs low fat male	11.8*	2.6
High muscle vs low muscle male	1.2	8.9*
High fat vs low fat female	12.0**	−1.5
High muscle vs low muscle female	0.7	7.4*

*p < 0.05, **p < 0.01.

## References

[b1] Cash T. F. & Deagle E. A. 3rd. The nature and extent of body-image disturbances in anorexia nervosa and bulimia nervosa: a meta-analysis. Int J Eat Disord 22, 107–125 (1997). 9261648

[b2] Gardner R. M. Methodological issues in assessment of the perceptual component of body image disturbance. Br J Psychol 87 (Pt 2), 327–337 (1996). 8673361 10.1111/j.2044-8295.1996.tb02593.x

[b3] Quick V. . Body size perception and weight control in youth: 9-year international trends from 24 countries. Int J Obes (Lond) 38, 988–994, doi: 10.1038/ijo.2014.62 (2014). 24722544 PMC4090285

[b4] Gunnare N. A., Silliman K. & Morris M. N. Accuracy of self-reported weight and role of gender, body mass index, weight satisfaction, weighing behavior, and physical activity among rural college students. Body Image 10, 406–410, doi: 10.1016/j.bodyim.2013.01.006 (2013). 23419637

[b5] Martin B. C. . Weight status misperception as related to selected health risk behaviors among middle school students. J Sch Health 84, 116–123, doi: 10.1111/josh.12128 (2014). 25099426

[b6] Powell T. M. . Body size misperception: a novel determinant in the obesity epidemic. Arch Intern Med 170, 1695–1697, doi: 10.1001/archinternmed.2010.314 (2010). 20937931 PMC4874507

[b7] Shin A. & Nam C. M. Weight perception and its association with socio-demographic and health-related factors among Korean adolescents. BMC Public Health 15, 1292, doi: 10.1186/s12889-015-2624-2 (2015). 26703247 PMC4690271

[b8] Yaemsiri S., Slining M. M. & Agarwal S. K. Perceived weight status, overweight diagnosis, and weight control among US adults: the NHANES 2003-2008 Study. International Journal of Obesity 35, 1063–1070, doi: 10.1038/ijo.2010.229 (2011). 21042327

[b9] Mond J. . Quality of life impairment associated with body dissatisfaction in a general population sample of women. BMC Public Health 13, 920, doi: 10.1186/1471-2458-13-920 (2013). 24088248 PMC3850528

[b10] van den Berg P. A., Mond J., Eisenberg M., Ackard D. & Neumark-Sztainer D. The Link Between Body Dissatisfaction and Self-Esteem in Adolescents: Similarities Across Gender, Age, Weight Status, Race/Ethnicity, and Socioeconomic Status. J Adolescent Health 47, 290–296, doi: 10.1016/j.jadohealth.2010.02.004 (2010). PMC2923488 20708569

[b11] Sutin A. R. & Terracciano A. Body weight misperception in adolescence and incident obesity in young adulthood. Psychol Sci 26, 507–511, doi: 10.1177/0956797614566319 (2015). 25749701

[b12] Preston C. & Ehrsson H. H. Illusory Changes in Body Size Modulate Body Satisfaction in a Way That Is Related to Non-Clinical Eating Disorder Psychopathology. PLoS ONE 9, e85773, doi: 10.1371/journal.pone.0085773 (2014). 24465698 PMC3897512

[b13] Paul T. K. . Size misperception among overweight and obese families. Journal of General Internal Medicine 30, 43–50, doi: 10.1007/s11606-014-3002-y25223750 (2015). 25223750 PMC4284259

[b14] Barlett C. P., Vowels C. L. & Saucier D. A. Meta-analyses of the effects of media images on men’s body-image concerns. Journal of Social and Clinical Psychology 27, 279–310, doi: 10.1521/jscp.2008.27.3.279 (2008).

[b15] Levine M. P. & Murnen S. K. “Everybody knows that mass media are/are not. Journal of Social and Clinical Psychology. 28, pp, doi: 10.1521/jscp.2009.28.1.9 (2009).

[b16] Robinson E. & Kirkham T. C. Is he a healthy weight? Exposure to obesity changes perception of the weight status of others. International Journal of Obesity 38, 663–667, doi: 10.1038/ijo.2013.15423949613 (2014). 23949613

[b17] Brooks K. R., Mond J. M., Stephen I. D. & Stevenson R. J. Body Image Distortion and Exposure to Extreme Body Types: Contingent Adaptation and Cross Adaptation for Self and Other. Frontiers in Neuroscience 10, 334, doi: 10.3389/fnins.2016.00334 (2016). 27471447 PMC4946181

[b18] Glauert R., Rhodes G., Byrne S., Fink B. & Grammer K. Body dissatisfaction and the effects of perceptual exposure on body norms and ideals. Int J Eat Disord 42, 443–452, doi: 10.1002/eat.20640 (2009). 19115365

[b19] Mohr H. M., Rickmeyer C., Hummel D., Ernst M. & Grabhorn R. Altered Visual Adaptation to Body Shape in Eating Disorders: Implications for Body Image Distortion. Perception. 725–738, doi: 10.1177/0301006616633385 (2016). 26921409

[b20] Rhodes G., Jeffery L., Boeing A. & Calder A. J. Visual coding of human bodies: perceptual aftereffects reveal norm-based, opponent coding of body identity. J Exp Psychol Hum Percept Perform 39, 313–317, doi: 10.1037/a0031568 (2013). 23398261

[b21] Winkler C. & Rhodes G. Perceptual adaptation affects attractiveness of female bodies. British Journal of Psychology 96, 141–154, doi: 10.1348/000712605x36343 (2005). 15969827

[b22] Frisby J. P. Seeing: illusion, brain, and mind. (Oxford University Press, 1980).

[b23] Blakemore C., Nachmias J. & Sutton P. The perceived spatial frequency shift: evidence for frequency-selective neurones in the human brain. J Physiol 210, 727–750 (1970). 5499822 10.1113/jphysiol.1970.sp009238 PMC1395609

[b24] Blakemore C. & Sutton P. Size adaptation: a new aftereffect. Science 166, 245–247 (1969). 5809598 10.1126/science.166.3902.245

[b25] Webster M. A. Adaptation and visual coding. Journal of Vision 11, doi: 10.1167/11.5.3 (2011). PMC3245980 21602298

[b26] Thompson P. & Burr D. Visual aftereffects. Current Biology 19, R11–R14 (2009). 19138580 10.1016/j.cub.2008.10.014

[b27] Russell J. A. & Fehr B. Relativity in the perception of emotion in facial expressions. Journal of Experimental Psychology: General 116, 223–237, doi: 10.1037/0096-3445.116.3.223 (1987).

[b28] O’Neil S. F. & Webster M. A. Adaptation and the perception of facial age. Vis cogn 19, 534–550, doi: 10.1080/13506285.2011.561262 (2011). 22215952 PMC3247904

[b29] Webster M. A., Kaping D., Mizokami Y. & Duhamel P. Adaptation to natural facial categories. Nature 428, 557–561, doi: 10.1038/nature02420 (2004). 15058304

[b30] Gwinn O. S. & Brooks K. R. Race-contingent face aftereffects: a result of perceived racial typicality, not categorization. Journal of vision 13, article 13, doi: 10.1167/13.11.13 (2013). 23970436

[b31] Gwinn O. S. & Brooks K. R. Face encoding is not categorical: Consistent evidence across multiple types of contingent aftereffects. Visual Cognition 23, 867–893, doi: 10.1080/13506285.2015.1091800 (2015).

[b32] Gwinn O. S. & Brooks K. R. No role for lightness in the encoding of Black and White: Race-contingent face aftereffects depend on facial morphology, not facial luminance. Visual Cognition 23, 597–611, doi: 10.1080/13506285.2015.1061085 (2015).

[b33] Carbon C. & Ditye T. Face adaptation effects show strong and long-lasting transfer from lab to more ecological contexts. Frontiers in psychology 3, doi: 10.3389/fpsyg.2012.00003 (2012). PMC3264890 22291676

[b34] Rhodes G., Jeffery L., Watson T. L., Clifford C. W. & Nakayama K. Fitting the mind to the world: face adaptation and attractiveness aftereffects. Psychol Sci 14, 558–566 (2003). 14629686 10.1046/j.0956-7976.2003.psci_1465.x

[b35] Barlow H. B. & Hill R. M. Selective sensitivity to direction of movement in ganglion cells of the rabbit retina. Science 139, 412–414 (1963). 13966712 10.1126/science.139.3553.412

[b36] Hummel D. . Neural adaptation to thin and fat bodies in the fusiform body area and middle occipital gyrus: an fMRI adaptation study. Hum Brain Mapp 34, 3233–3246, doi: 10.1002/hbm.22135 (2013). 22807338 PMC6870049

[b37] Hummel D., Rudolf A. K., Untch K. H., Grabhorn R. & Mohr H. M. Visual adaptation to thin and fat bodies transfers across identity. PLoS One 7, e43195, doi: 10.1371/journal.pone.0043195 (2012). 22905232 PMC3419644

[b38] Palumbo R., D’Ascenzo S. & Tommasi L. Cross-category adaptation: exposure to faces produces gender aftereffects in body perception. Psychol Res 79, 380–388, doi: 10.1007/s00426-014-0576-2 (2015). 24859840

[b39] Palumbo R., Laeng B. & Tommasi L. Gender-specific aftereffects following adaptation to silhouettes of human bodies. Visual Cognition 21, 1–12, doi: 10.1080/13506285.2012.753970 (2013).

[b40] Sekunova A., Black M., Parkinson L. & Barton J. J. Viewpoint and pose in body-form adaptation. Perception 42, 176–186 (2013). 23700956 10.1068/p7265

[b41] Stephen I. D. & Perera A. T. M. Judging the Difference between Attractiveness and Health: Does Exposure to Model Images Influence the Judgments Made by Men and Women? Plos One 9, doi: 10.1371/journal.pone.0086302 (2014). PMC3896486 24466014

[b42] Mele S., Cazzato V. & Urgesi C. The Importance of Perceptual Experience in the Esthetic Appreciation of the Body. PloS ONE 8, e81378, doi: 10.1371/journal.pone.0081378 (2013). 24324689 PMC3852268

[b43] Winkler C. & Rhodes G. Perceptual adaptation affects attractiveness of female bodies. British Journal of Psychology 96, 141–154, doi: 10.1348/000712605X36343 (2005). 15969827

[b44] Cazzato V. . The effects of body exposure on self-body image and esthetic appreciation in anorexia nervosa. Experimental Brain Research 234, 695–709 (2016). 26586269 10.1007/s00221-015-4498-z

[b45] Myint P. K., Kwok C. S., Luben R. N., Wareham N. J. & Khaw K. T. Body fat percentage, body mass index and waist-to-hip ratio as predictors of mortality and cardiovascular disease. Heart 100, 1613–1619, doi: 10.1136/heartjnl-2014-305816 (2014). 24966306

[b46] Schulze M. B. . Body adiposity index, body fat content and incidence of type 2 diabetes. Diabetologia 55, 1660–1667, doi: 10.1007/s00125-012-2499-z (2012). 22349074

[b47] Wannamethee S. G., Shaper A. G., Lennon L. & Whincup P. H. Decreased muscle mass and increased central adiposity are independently related to mortality in older men. Am J Clin Nutr 86, 1339–1346 (2007). 17991644 10.1093/ajcn/86.5.1339

[b48] Deurenberg-Yap M., Chew S. K. & Deurenberg P. Elevated body fat percentage and cardiovascular risks at low body mass index levels among Singaporean Chinese, Malays and Indians. Obes Rev 3, 209–215 (2002). 12164474 10.1046/j.1467-789x.2002.00069.x

[b49] Lee C. M., Huxley R. R., Wildman R. P. & Woodward M. Indices of abdominal obesity are better discriminators of cardiovascular risk factors than BMI: a meta-analysis. J Clin Epidemiol 61, 646–653, doi: 10.1016/j.jclinepi.2007.08.012 (2008). 18359190

[b50] Lawler M. & Nixon E. Body dissatisfaction among adolescent boys and girls: The effects of body mass, peer appearance culture and internalization of appearance ideals. Journal of Youth and Adolescence 40, 59–71, doi: 10.1007/s10964-009-9500-220058058 (2011). 20058058

[b51] Mitchison D. & Mond J. M. Epidemiology of eating disorders, eating-disordered behaviour, and body image disturbance in males: A narrative review. Journal of Eating Disorders 3, 9, doi: 10.1186/s40337-015-0058-y (2015). 27408719 PMC4940910

[b52] Murray S. B., Griffiths S. & Mond J. M. Evolving eating disorder psychopathology: conceptualising muscularity-oriented disordered eating. Br J Psychiatry 208, 414–415, doi: 10.1192/bjp.bp.115.168427 (2016). 27143005

[b53] Maida D. M. & Lee Armstrong S. The Classification of Muscle Dysmorphia. International Journal of Men’s Health 4, 73–91, doi: 10.3149/jmh.0401.73 (2005).

[b54] Olivardia R., Pope H. G. Jr. & Hudson J. I. Muscle dysmorphia in male weightlifters: a case-control study. Am J Psychiatry 157, 1291–1296, doi: 10.1176/appi.ajp.157.8.1291 (2000). 10910793

[b55] Benninghoven D., Raykowski L., Solzbacher S., Kunzendorf S. & Jantschek G. Body images of patients with anorexia nervosa, bulimia nervosa and female control subjects: A comparison with male ideals of female attractiveness. Body Image 4, 51–59, doi: 10.1016/j.bodyim.2006.12.00218089251 (2007). 18089251

[b56] Hartmann A. S., Greenberg J. L. & Wilhelm S. The relationship between anorexia nervosa and body dysmorphic disorder. Clinical Psychology Review 33, 675–685, doi: 10.1016/j.cpr.2013.04.00223685673 (2013). 23685673

[b57] Brierley M. E., Brooks K. R., Mond J., Stevenson R. J. & Stephen I. D. The Body and the Beautiful: Health, Attractiveness and Body Composition in Men’s and Women’s Bodies. PLoS One 11, e0156722, doi: 10.1371/journal.pone.0156722 (2016). 27257677 PMC4892674

[b58] Stephen I. D. & Perera A. T.-M. Judging the differences between women’s attractiveness and health: Is there really a difference between judgments made by men and women? Body Image 11, 183–186, doi: 10.1016/j.bodyim.2013.11.00724405818 (2014). 24405818

[b59] TanitaUKLtd. Body Composition Analyser SC-330: Instruction manual. (Tanita, 2008).

[b60] Tiddeman B., Burt M. & Perrett D. Prototyping and transforming facial textures for perception research. Ieee Comput Graph 21, 42–50, doi: 10.1109/38.946630 (2001).

[b61] Howell D. C. Statistical Methods for Psychology. (Duxbury Press, 1997).

[b62] Valentine T. A unified account of the effects of distinctiveness, inversion, and race in face recognition. Q J Exp Psychol A 43, 161–204 (1991). 1866456 10.1080/14640749108400966

[b63] Holland G. & Tiggemann M. “Strong beats skinny every time”: Disordered eating and compulsive exercise in women who post fitspiration on Instagram. International Journal of Eating Disorders, doi: 10.1002/eat.22559 (2016). 27302867

[b64] Benton C. & Karazsia B. T. The effect of thin and muscular images on women’s body satisfaction. Body image 13, 22–27 (2015). 25528369 10.1016/j.bodyim.2014.11.001

